# Interaction of benzo[a]pyrene diol epoxide isomers with human serum albumin: Site specific characterisation of adducts and associated kinetics

**DOI:** 10.1038/srep36243

**Published:** 2016-11-02

**Authors:** Hitesh V. Motwani, Emelie Westberg, Margareta Törnqvist

**Affiliations:** 1 Department of Environmental Science and Analytical Chemistry, Stockholm University, SE-10691 Stockholm, Sweden

## Abstract

Carcinogenicity of benzo[a]pyrene {B[a]P, a polycyclic aromatic hydrocarbon (PAH)} involves DNA-modification by B[a]P diol epoxide (BPDE) metabolites. Adducts to serum albumin (SA) are not repaired, unlike DNA adducts, and therefore considered advantageous in assessment of *in vivo* dose of BPDEs. In the present work, kinetic experiments were performed in relation to the dose (i.e. concentration over time) of different BPDE isomers, where human SA (hSA) was incubated with respective BPDEs under physiological conditions. A liquid chromatography (LC) tandem mass spectrometry methodology was employed for characterising respective BPDE-adducts at histidine and lysine. This strategy allowed to structurally distinguish between the adducts from racemic *anti*- and *syn*-BPDE and between (+)- and (−)-*anti*-BPDE, which has not been attained earlier. The adduct levels quantified by LC-UV and the estimated rate of disappearance of BPDEs in presence of hSA gave an insight into the reactivity of the diol epoxides towards the N-sites on SA. The structure specific method and dosimetry described in this work could be used for accurate estimation of *in vivo* dose of the BPDEs following exposure to B[a]P, primarily in dose response studies of genotoxicity, e.g. in mice, to aid in quantitative risk assessment of PAHs.

Polycyclic aromatic hydrocarbons (PAHs), several of which are carcinogens, are formed during incomplete combustion of organic matters and are known to be ubiquitous in the environment. Exposure of the general population to PAHs occurs from food and other sources such as cigarette smoke, vehicle exhausts and domestic wood burning^1^,^2^,^3^. Food can be contaminated by PAHs from the environment or during preparation, e.g. when smoked or grilled^4^,^5^.

Benzo[a]pyrene (B[a]P), classified as a human carcinogen (IARC 2012)^2^, has widely been used as an indicator of carcinogenic PAHs, and is considered one of the most prevalent and carcinogenic of PAHs^3^. Metabolism of B[a]P gives rise to the electrophilic reactive B[a]P diol epoxides (BPDEs, Fig. 1)^6^,^7^: (+)- and (−)-*anti*−7β,8α-dihydroxy-9α,10α-epoxy-7,8,9,10-tetrahydrobenzo[a]pyrene (*anti*-BPDE), and (+)- and (−)-*syn*−7β,8α-dihydroxy-9β,10β-epoxy-7,8,9,10-tetrahydrobenzo[a]pyrene (*syn*-BPDE). The diol epoxides are the causative factors responsible for DNA damage from B[a]P-exposure, and amongst them the most potent mutagenic and carcinogenic metabolite has been identified as (+)-*anti*-BPDE^8^. The BPDE stereoisomers covalently bind with varying degree of reactivity to DNA, e.g. to exocyclic amino groups on deoxyguanosine, resulting in formation of DNA adducts^7^.

Adducts formed through covalent binding of low molecular weight electrophilic compounds to nucleophilic sites on blood proteins are often used as a surrogate biomarker for the corresponding DNA adducts^9^. Analysis of adducts to haemoglobin (Hb) and serum albumin (SA) have, compared to DNA adducts, advantages of high abundance of the proteins. Unlike DNA, damage to the protein is not repaired and hence protein adducts have an inherent advantage as a measure for human exposure, e.g. in epidemiologic studies^9^,^10^. Hb adducts have been used for measurement of dose of the precursor electrophile in blood, i.e. referred as the *in vivo* dose or “the area under the concentration-time curve” (AUC), which can be used as a basis in cancer risk assessment^11^,^12^,^13^. However, with regard to adducts from diol epoxides of PAHs there is no sufficiently established method for protein adduct measurements that can be used for accurate measurement of the *in vivo* dose.

Albumin is the most abundant protein in serum. Albumin, possessing large hydrophobic pockets, is involved in the transport of various endogenous substrates as well as lipophilic xenobiotics^14^,^15^. SA adducts of BPDEs have been studied with different methods (reviewed in Boysen and Hecht, 2003^16^ and Kafferlein *et al*.^17^). One approach to measure the BPDE adducts to SA or Hb is to detach the adduct by hydrolysis to form corresponding tetrols, which are then detected by gas chromatography (GC) or liquid chromatography (LC) in combination with fluorescence or mass spectrometry (MS). Carboxylic ester adducts in albumin with aspartate and glutamate, considered favourable for binding the (+)-*anti*-BPDE^18^, could be released as tetrols following mild acidic hydrolysis *in vitro*. This approach has shown to be sensitive enough for application to studies of PAH-exposed humans, for instance occupationally exposed workers^17^. Disadvantages are though that the measured hydrolysed products have no tag from the protein proving that they correspond to released adducts and the analytes also lack specificity with regard to the binding site of BPDE in the protein. Moreover, the ester adducts are prone to undergo hydrolysis also *in vivo.* These circumstances make it difficult to calculate dose *in vivo* from measured tetrols and they are therefore not suitable as biomarkers for *in vivo* dose assessment. Further, currently available immunoassays to measure BPDE-adducts from SA provides a measure for general PAH exposure rather than specifically for B[a]P, due to cross-reactivity of the antibody with other PAHs^17^.

Adducts to N-sites of amino acids, such as histidine and lysine, are more stable compared to the ester adducts. Indeed, His^146^ and Lys^195^ have been assigned as the sites on SA for binding of BPDEs^19^. In our earlier work, methods to measure histidine adducts from (±)-*anti*-BPDE in human SA (hSA) were studied. One approach tested was to cleave the albumin by hydrazinolysis and detect the histidine hydrazide adduct by LC tandem mass spectrometry (MS/MS)^20^, though the approach was considered difficult to use for routine procedure due to the hazardous properties of hydrazine. Recently, we developed a method for LC-MS/MS measurement of histidine and lysine adducts from (±)-*anti*-BPDE in hSA based on enzymatic digestion^21^. Even though structural information was obtained from this study, separation of the (+)- and (−)-enantiomer adducts of *anti*-BPDE was not attained that could be useful to assess the relative reactivity of each enantiomer and to confirm their stereospecific conformation. Furthermore, the adduct formation from the *syn*-form is not well-characterised, and, thus there is a lack of knowledge about the extent of binding and reactivity of the *syn*-BPDEs towards the nucleophilic sites of SA. Consequently, so far it has not been possible to use these methods in their present form to measure *in vivo* dose of the individual BPDE isomers, e.g. in mice.

In the present study we characterise the formation of histidine- and lysine-adducts from (±)-*anti*-, (+)-*anti*- and (±)-*syn*-BPDE in hSA by a structure specific LC-MS/MS methodology. Formed adduct levels and disappearance of respective BPDEs over time were measured under physiological conditions to obtain the rate of adduct formation and relationship between adduct level in hSA and dose (i.e. concentration over time) for the different BPDE isomers. This relationship could be applied in future *in vivo* studies to estimate the AUC of BPDEs from measured adduct levels of the diol epoxides arising from B[a]P exposure. The *in vivo* doses of electrophilic metabolites are potentially useful as a quantitative tool to facilitate species-extrapolation of genotoxic effects and cancer risk from animal studies to human exposure.

## Results

### Characterisation of serum albumin adducts to BPDEs

Human serum albumin was incubated *in vitro* under physiological conditions with (±)-*anti*-, (+)-*anti*- and (±)-*syn*-BPDE; albumin to BPDE molar ratio of 9:1. Following the incubation period (*t*) for 24 h, the protein was precipitated and enzymatically digested by pronase, and subsequently the adducts were enriched by solid phase extraction (SPE) and characterised by LC-MS/MS. A recently developed MS/MS method for analysis of the albumin adducts [with a limit of detection of about 1 fmol adduct/mg SA for (±)-*anti*-BPDE^21^] was adopted to measure the generated analytes, BPDE-His-Pro, BPDE-His-Pro-Tyr and BPDE-Lys. However, the tripeptide was not detected in any of the samples, probably due to a near complete digestion of the protein by pronase^22^,^23^.

**Figure 2:**
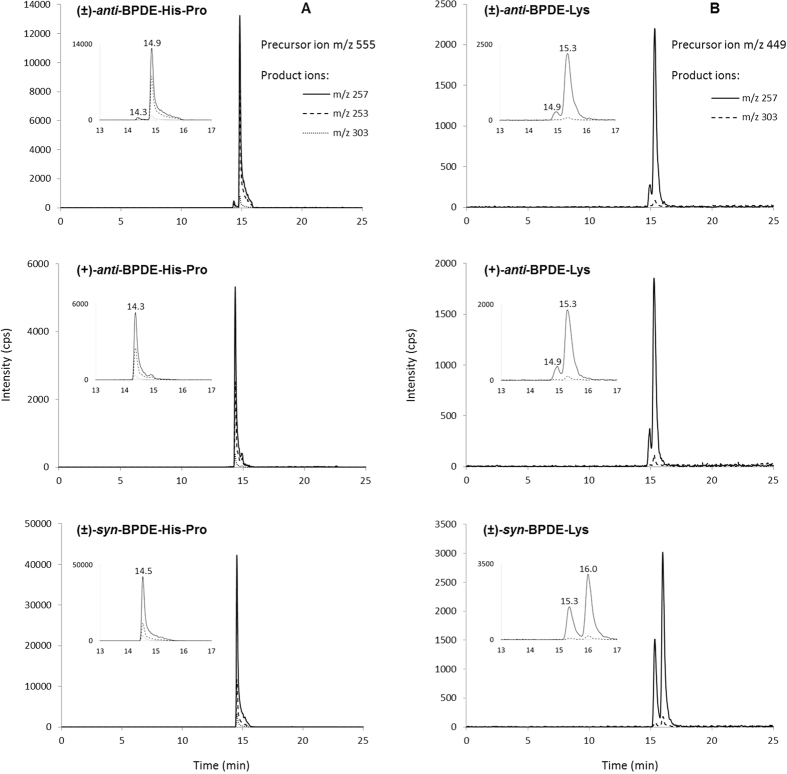
LC-MRM chromatograms showing peaks of BPDE-His-Pro (**A**) and BPDE-Lys (**B**) formed from hSA alkylated with (±)-*anti*-, (+)-*anti*- and (±)-*syn*-BPDE. Region between 13–17 min is inserted in each chromatogram.

The LC-MS/MS (MRM) chromatograms in Fig. 2 show BPDE-His-Pro and BPDE-Lys from the respective incubation studies with (±)-*anti*-BPDE, (+)-*anti*-BPDE and (±)-*syn*-BPDE. LC conditions were adjusted to separate analytes from the *anti*- and *syn*-isomers, and between the enantiomers of each. This resulted in developing two setup of LC conditions, which are described in Methods. Setup 1 was applied to separate histidine analytes formed from the *anti*-enantiomers, (+) and (−); with BPDE-His-Pro from (+)-*anti*-BPDE (at 14.3 min) eluting nearly half a minute before that from (−)-*anti*-BPDE (at 14.9 min), and the His-Pro analyte from (±)-*syn*-BPDE eluting between the analytes from (+) and (−)-*anti*-BPDE (Fig. 2 and Table 1). With regard to the BPDE-Lys analytes (setup 2), a complete separation was not obtained between those from the respective enantiomers, requiring further optimisation of the LC conditions if this would be desired.

The MS-method using ESI^+^ was effective in determining site of adduct formation, in this case to histidine or lysine, and peptide length (i.e. mono- and dipeptide) following digestion of the protein (Fig. 2 and Table 1). Table 1 summarizes the characterisation of the measured BPDE-adducts on histidine and lysine in SA, with retention times, the precursor and product ions used and relative intensities. The fragmentation pattern was in agreement with that described for the albumin adducts from (±)-*anti*-BPDE^21^, with m/z 257 as the predominant fragment from BPDE-His-Pro and BPDE-Lys (cf. Westberg *et al*.^21^ for proposed structures of the fragments).

### Measurement of the adduct levels

**Figure 3:**
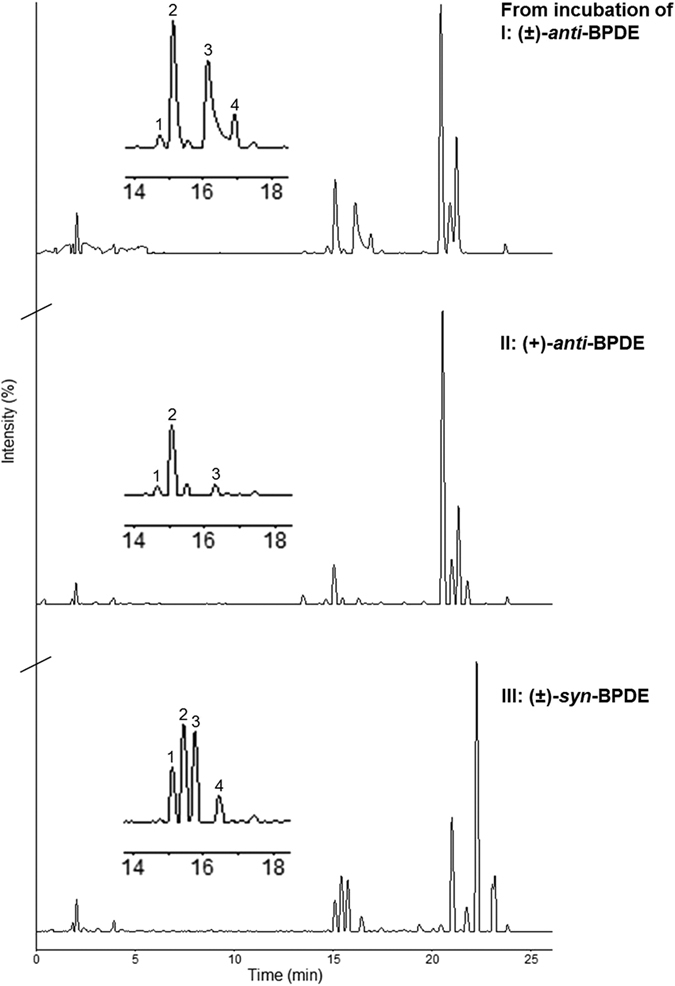
LC-UV chromatograms from incubation of (±)-*anti*-BPDE (I), (+)-*anti*-BPDE (II) and (±)-*syn*-BPDE (III) with hSA, at 37 °C, pH 7.5 for 24 h followed by enzymatic digestion of the albumin and isolation of the adducts; insert showing the region between 14–18 min. Where, peaks **1** and **2** correspond to BPDE-Lys, peak **3** to BPDE-His-Pro, and some unidentified peaks, e.g. peak 4, with array of peaks around 20–25 min from hydrolysed BPDEs e.g. tetrols. (Peaks 1, 2 and 3 were confirmed by MS/MS characterisation.)

Histidine and lysine adduct levels formed in hSA (0.6 mM) from (±)-*anti*-BPDE, (+)-*anti*-BPDE and (±)-*syn*-BPDE [initial concentration (*C*_*0*_) of the different BPDEs were the same, 0.066 mM; *t* 24 h], following the digestion and isolation, were measured by LC-UV at 345 nm. Fig. 3 shows the chromatograms of respective adducts from the different BPDE isomers. The LC-UV system was coupled on-line to the mass spectrometer and peaks of BPDE-His-Pro and BPDE-Lys were confirmed based on their respective MS/MS spectra as described above. Additionally, there were minor unidentified peaks, e.g. peak 4 in Fig. 3. The respective adduct levels of BPDE-His-Pro (*A*_*His-Pro*_) and BPDE-Lys (*A*_*Lys*_), given in Table 2, were calculated from the area of the corresponding peaks in Fig. 3 and using equation (1) as described in Methods.

Adduct levels, *A*_*His-Pro*_ and *A*_*Lys*_, from the corresponding racemic (±)-*anti*- and (±)-*syn*-BPDE were similar, in the range of 1.3–1.9 pmol adduct/mg SA (Table 2). The (−)-*anti*-enantiomer of BPDE formed nearly 9 times more histidine adducts than the most potent mutagenic enantiomer, (+)-*anti*-BPDE, similar to that indicated earlier^18^,^24^. Conversely, it was found that the selectivity for adduct formation was reversed for the lysine site, with (+)-*anti*-enantiomer forming ca. 3 times more adducts than the (−)-*anti*-enantiomer as seen from Table 2.

### Kinetics of BPDEs in presence of albumin

**Figure 4:**
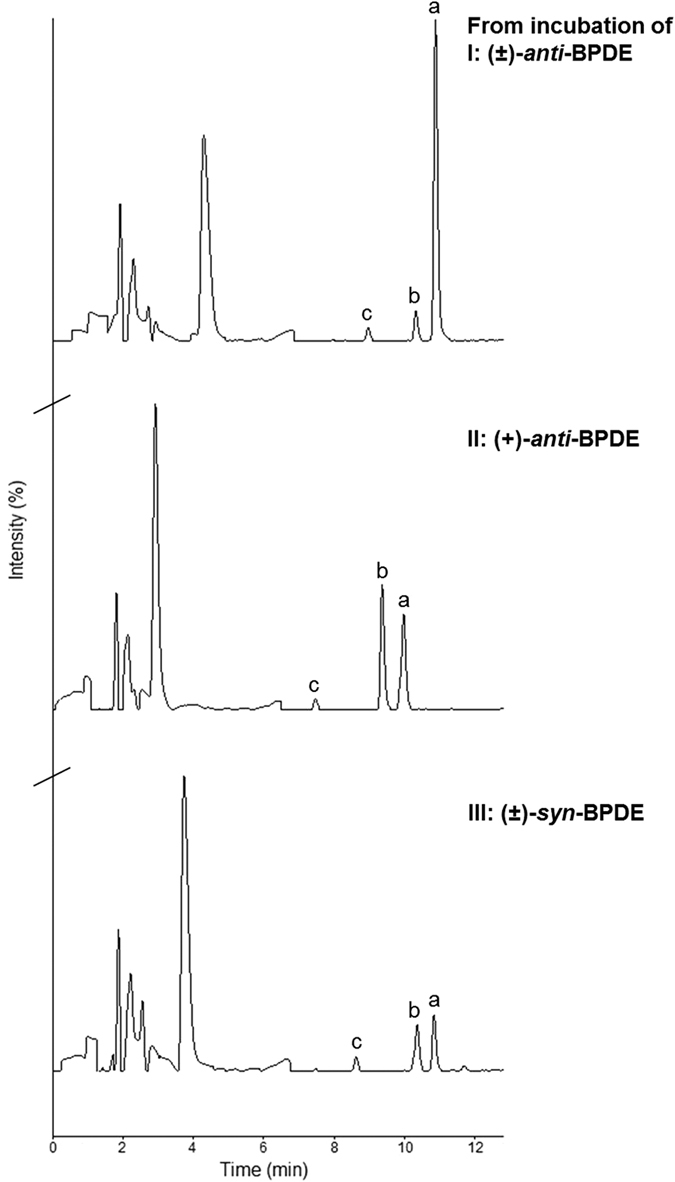
LC-UV chromatograms from incubation of (±)-*anti*-BPDE (I), (+)-*anti*-BPDE (II) and (±)-*syn*-BPDE (III) with hSA, at 37 °C, pH 7.5 for 20 min and subsequent derivatisation by BME. Where, peak **a** corresponds to BPDE-BME derivative, peaks **b** and **c** to hydrolysed products of BPDEs, as tetrols, and array of peaks before 5 min probably from polar components of the albumin. (Peak **a** in chromatograms I, II and III was confirmed by comparison with standard BPDE-BME formed under similar conditions. In II there was shift in retention time towards left since it was performed at a different occasion.)

BPDEs in aqueous mixture of SA, besides their reactions with the nucleophilic sites of the albumin, can also hydrolyse to tetrols, triols and ketones^25^,^26^. Kinetics of the studied diol epoxides (±)-*anti*-, (+)-*anti*- and (±)-*syn*-BPDE were investigated in the present study by determining their respective apparent disappearance rate under the incubation conditions used for measurement of the adduct levels. For this purpose, aliquots were taken at certain time intervals from the incubation mixture, the BPDE in the aliquots was derivatised by β-mercaptoethanol (BME), and subsequently analysed by LC-UV at 345 nm. Representative LC-UV chromatograms from incubation at 20 min are shown in Fig. 4. Extraction efficiency of recovering BPDE-BME from the hSA mixture was estimated >90%.

**Figure 5:**
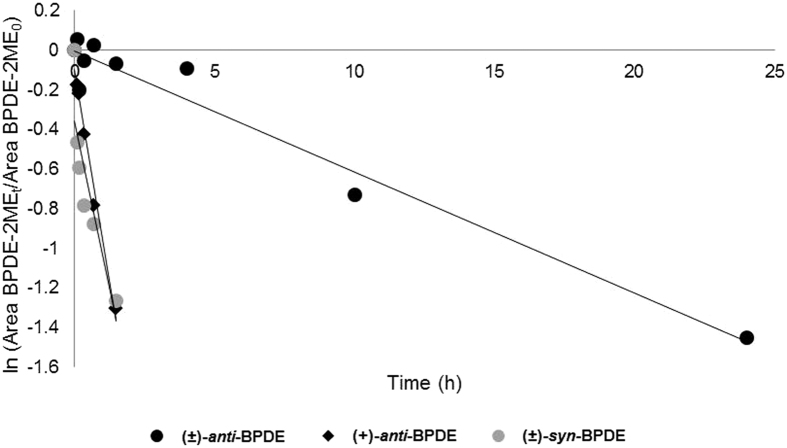
Plot showing disappearance of BPDEs in hSA, at 37 °C, pH 7.5. Values of *n* and *r*^*2*^ given in Table 2.

The disappearance rate of the BPDEs in hSA was measured by following the thiol derivative of BPDE, i.e. BPDE-BME (peak **a** in Fig. 4). Pseudo-first order rate constants for disappearance (*λ*) of the diol epoxides were determined by plotting peak area of BPDE-BME at a given time (Area BPDE-BME_t_) in relation to the area at time 0 min (Area BPDE-BME_0_) versus the corresponding incubation time as shown in Fig. 5; and the obtained values of apparent *λ* from the respective slopes are given in Table 2. Thereby the corresponding second-order rate constants calculated from concentration of the albumin (6 × 10^−4^ M) for disappearance of (±)-*anti*-, (+)-*anti*- and (±)-*syn*-BPDE are 0.028, 0.39 and 0.31, in M^−1^·s^−1^, respectively. Earlier, hydrolysis rate of (±)-*anti*- and (±)-*syn*-BPDE in albumin-free water at 25 °C have been reported as 4.3 × 10^−4^ s^−1^ and 1.5 × 10^−2^ s^−1^, respectively^26^,^27^. Altogether, these results show that the BPDEs are stabilized in presence of albumin, probably by formation of a physical association complex (BPDE-SA) as suggested earlier^26^,^28^,^29^. Islam *et al*. estimated the apparent equilibrium constant for association of (±)-*anti*-BPDE to hSA as 2–3 times greater than that of (±)-*syn*-BPDE^26^.

The *λ* value of (+)-*anti*-BPDE was ca. 14 times higher than (±)-*anti*-BPDE (Table 2), implying that under the incubation conditions the disappearance of the (−)-enantiomer was slower than that of the (+)-form. Further, the disappearance rates in combination with the adduct levels presented in Table 2 give an insight into the reactivity of the BPDEs towards the nucleophilic sites of the albumin. For instance, after 24 h reaction at same initial concentrations, the sum of the adduct levels to histidine and lysine (*A*_*His-Pro*_ + *A*_*Lys*_) from (±)-*anti*-BPDE and (±)-*syn*-BPDE were similar (in the range 2.8–3.2 pmol adduct/mg SA), despite that (±)-*syn*-BPDE eliminates ca. 11 times faster than the *anti-*form. This indicates that *syn*-BPDE could be considered to have a higher reactivity than the *anti*-form towards the N-sites of SA.

### Dosimetry of BPDEs

From the *in vitro* incubation performed with hSA, where *C*_*0*_ of BPDE was 0.066 mM, *t* 24 h and *λ* from Table 2, the dose *D* in mM·h of the BPDE isomers calculated using equation (3) (cf. Törnqvist *et al*.^9^) are given in Table 3. The *D* of BPDEs gives a measure of the concentration of the respective diol epoxides over time; referred to as AUC in *in vivo* situation^9^,^13^. Using the measured levels of the adducts to histidine and lysine (*A*_*His-Pro*_ and *A*_*Lys*_, Table 2) and *D*, the second-order rate constants for the reaction of BPDE isomers with the nucleophilic sites, histidine and lysine (*k*_*His-Pro*_ and *k*_*Lys*_, respectively), in SA were calculated [equation (4)] and are included in Table 3.

It is seen from Table 3 that in general the measured reactivity of the albumin’s N-sites is about a magnitude higher towards (±)-*syn*-BPDE than towards the (±)-*anti*-form under the applied conditions. The *k*_*His-Pro*_ and *k*_*Lys*_ values in the table show that lysine is more prone to react with (+)-*anti*-BPDE in comparison to histidine site of the protein. The selectivity between the two N-sites is nullified for the racemic form.

## Discussion

Measurement of adducts to blood proteins offers possibility to detect and quantify exposure of the precursor compound and to estimate the *in vivo* dose of the carcinogenic metabolites^9^,^30^. A prerequisite for this is to have accurate structure-specific analytical methods, which could be obtained by MS. It is preferred if the analyte includes a tag from the protein, as this would confirm that the analyte is originating from a covalent bound adduct. The characterisation of adduct formation to hSA from BPDE isomers by LC-MS/MS in the present investigation is an important advancement towards this approach for PAHs.

The carrier protein hSA has been associated with binding, stabilizing and transporting of bulky epoxides^15^,^26^,^28^. Most of the earlier work on studies of BPDE-induced changes of SA have been on the racemic *anti*-isomers (as in references^18^,^20^,^21^,^22^,^31^), with only a few studies on the *syn*-forms (mainly by Islam and co-workers^25^,^26^). With regard to specific MS methods for adduct characterisation, histidine and lysine adducts from racemic *anti*-BPDE have been resolved earlier by our group^21^,^24^, but we found no studies on characterisation of adducts from the *syn*-form. Histidine adduct of (+)-*anti*-BPDE from mouse SA was also measured^24^, but not with hSA and the two enantiomers of the *anti*-form were not separable in that study.

In the present work, a state-of-the-art MS methodology was used to characterise modification induced by BPDE isomers on SA at histidine and lysine. SA adduct levels formed during physiological conditions in hSA from the various BPDEs at the same initial concentrations were quantified (Table 2). The varying selectivity observed of the protein adduct formation could be explained by a preceding noncovalent binding, which would control rotational and translational movement of the BPDE^22^,^29^. Other studies on different PAHs epoxy-metabolites have also shown selective alkylation of the albumin sites, e.g. binding by different diol epoxides isomers of fluoranthene, chrysene, benz[a]anthracene and benzo[c]phenanthrene to histidine or lysine^22^,^32^. From kinetic experiments it was indicated that in presence of SA, the BPDE isomers are protected from hydrolysis by varying degrees depending upon their conformation (cf. Table 2), probably by entering the hydrophobic pockets of the albumin^26^,^28^. Further, the concentration over incubation time, i.e. dose *D*, of BPDEs in the hSA mixture was estimated and used to calculate the rate of formation of the adducts (Table 3). From the same initial concentration of *anti*-BPDE enantiomers, the dose *D* of the most potent mutagenic metabolite of B[a]P, (+)-*anti*-BPDE, is expected to be about an order of magnitude lower than the (−)-enantiomer (cf. *λ* in Table 2 and *D* in Table 3). The results overall give a mechanistic insight into the relative reactivity of the diol epoxides towards N-sites of the protein.

The rate of adduct formation to histidine of SA from the different isomers can be expressed as 1.7–13 pmol/g SA per μM·h to simplify comparisons (from Table 3). This is much lower than the rate of adduct formation to DNA; earlier studies of adduct levels in human blood after *in vitro* reaction with (±)-*anti*-BPDE showed about 10^3^ times higher adduct levels to deoxyguanosine in DNA than to histidine in SA^24^. Though, the reactivity of BPDE towards the nucleophilic sites in SA is of the same magnitude as that of low molecular weight genotoxic epoxides towards N-terminal valine in Hb^33^. Such adducts to Hb are established for monitoring of *in vivo* dose, or AUC, of e.g. glycidamide, genotoxic metabolite of acrylamide from dietary exposure in humans^33^.

The present study gives results that have a potential to be applied in an approach that uses albumin adduct levels to estimate *in vivo* doses of metabolites of B[a]P as an indicator compound for PAHs. Primarily, the *in vivo* dosimetry will be used in our future work to estimate genotoxicity per dose unit of diol epoxide metabolites of B[a]P *in vivo*, for comparison with other carcinogens as well as with genotoxic potency *in vitro*^11^,^12^. It might not be possible to measure the protein-adduct specifically from the (+)-*anti*-BPDE enantiomer in humans, e.g. from exposure via food, with the current available methods. In such case an inter-species comparison of the doses (AUC) of the carcinogenic metabolites in humans might be obtained from *in vivo* doses in exposed animals and metabolism studies through a parallelogram approach (cf. Motwani and Törnqvist, 2014^34^). The derived *in vivo* doses based on the albumin adducts can then further be applied in different procedures to aid in quantitative cancer risk estimation of PAHs.

## Methods

### Chemicals and other materials used

β-Mercaptoethanol (BME, ≥ 99%) was obtained from Sigma-Aldrich (Steinheim, Germany). (±)-*anti*-, (±)-*syn*- and (+)-*anti*-Benzo[a]pyrene-7,8-diol-9,10-epoxide (BPDE) were purchased from the National Cancer Institute (NCI), Chemical Carcinogen References Standard Repository (Kansas City, KS, USA). Purity of (±)-*anti*-, (±)-*syn*- and (+)-*anti*-BPDE was ca. 95%, 95% and 80%, respectively, determined by derivatisation with BME (cf. Michaud *et al*.^35^). Stock solutions of the three BPDEs (each 10 μg/μL) were prepared in ice-cold tetrahydrofuran (500 μL). Lyophilised human serum albumin (hSA) and pronase from *Streptomyces griseus* were obtained from Sigma-Aldrich (Steinheim, Germany). All solvents and chemicals used were of HPLC and analytical grade, respectively. Sep-Pak Plus C_18_ solid phase extraction (SPE) cartridges (360 mg) were purchased from Waters (Milford, MA, USA). Eppendorf Protein LoBind tubes (Hamburg, Germany) were used for albumin samples.

*Caution*. BPDEs are known carcinogenic compounds. They were handled carefully in a well-ventilated fume-hood and destroyed immediately after use by 1 M aqueous H_2_SO_4_.

### Incubation of hSA with (±)-*anti*-BPDE, (+)-*anti*-BPDE and (±)-*syn*-BPDE

BPDE solution in tetrahydrofuran was added to hSA (80 mg) in ice-cold water (2 mL) to give a final solution of 0.5 μg BPDE per mg hSA, and the pH was adjusted to 7.5 with NaOH (0.1 M, ca. 10 μL) as previously described^21^. The mixture was incubated at 37 °C for 24 h in a water-bath. Incubation with each of the three BPDE isomers was performed to characterise and measure the SA adducts, and to estimate the apparent disappearance rate constants as described below.

### Kinetic studies of BPDE in hSA

Aliquots (200 μL) were taken out from the incubation at certain time intervals and mixed with BME (2 M in 0.4 M aq. NaOH, 500 μL) for 30 min at room temperature to form the BME derivative of BPDE (BPDE-BME). The mixture was cooled on ice-bath and centrifuged (14000 rpm, 4 °C, 20 min). The supernatant was extracted with ethyl acetate (400 μL × 3), washed with water (200 μL × 2), and the organic phase was dried under N_2_. Methanol-water (1:1 v/v, 400 μL) was added to the samples and subsequently the respective BPDE-BME derivatives from (±)-*anti*-BPDE, (+)-*anti*-BPDE and (±)-*syn*-BPDE were analysed by LC-UV. Using the area from the LC-UV measurements, ln (Area BPDE-BME_t_/Area BPDE-BME_0_) versus the corresponding incubation time was plotted to determine the disappearance rate constant *λ* for the BPDE isomers. Values of *n* and *r*^*2*^ for the individual plots are given in Table 2. Standard deviation (SD) of the slope, i.e. *λ*, was calculated using the Excel array function LINEST. The experiment using (±)-*anti*-BPDE with 20 min incubation time was repeated thrice, all giving similar peak area of BPDE-BME by LC-UV as shown in Fig. 4.

### Extraction efficiency to recover BPDE-BME

(±)-*anti*-BPDE was used in a test to estimate the extraction efficiency (in %) of BPDE-BME following centrifugation and extraction as described above in kinetic studies. In three parallel experiments, free BPDE (4 μg in 200 μL), i.e. without albumin, was prepared from the stock solution in ice-cold water. This was added to BME (2 M in 0.4 M aq. NaOH, 500 μL) and the reaction was continued for 30 min. The aqueous samples were analysed by LC-UV and the area of BPDE-BME peak was compared with those from the kinetic study of (±)-*anti*-BPDE in hSA at 0 min.

### Standard solution of BPDE derivatised with BME

Standard BPDE-BME derivative was prepared by a similar method as described earlier^35^. An alkaline solution of BME (2 M, 10 mL) was prepared using 0.4 M aq. NaOH. Under ice-cold conditions water was added to a stock solution of respective BPDE in tetrahydrofuran (10 μg/μL) to give a concentration of 20 μg/mL. Two hundred microliter from this solution of BPDE was mixed with 500 μL of the alkaline BME solution for 30 min at room temperature. The mixture was extracted with ethyl acetate (400 μL × 3), washed with water (200 μL × 2), and the organic phase dried under N_2_ to give BPDE-BME, which was diluted by methanol-water (1:1 v/v, 400 μL) and analysed by LC-UV.

### Isolation of BPDE-adducts of hSA

Alkylation of hSA (80 mg) in 2 mL water with respective BPDEs was investigated following the incubation experiments described above at 37 °C, pH 7.5 for 24 h, with a concentration of 0.5 μg BPDE per mg hSA. The resulting adducts were isolated according to an earlier described method^21^. Briefly, following incubation after 24 h, the mixture was adjusted to pH 4 with 1 M HCl and a saturated solution of ammonium sulphate (2 mL) was added to precipitate the BPDE-alkylated hSA, at ca. 4 °C for 30 min. The precipitate was isolated, washed with methanol (10 mL), ethyl acetate (10 mL × 2) and pentane (5 mL) and dried at room temperature. Subsequently, for enzymatic hydrolysis BPDE-alkylated hSA (10 mg) was mixed with pronase (0.9 mg) in aq. ammonium bicarbonate (50 mM, 450 μL), and the mixture was incubated at 37 °C for ca. 20 h. The obtained BPDE-alkylated peptide analytes were isolated by SPE and concentrated to 150 μL in water, and analysed by LC-UV and LC-MS/MS.

### LC-UV and LC-MS/MS

A tandem LC-UV-MS/MS system that consisted of a Shimadzu Prominence LC 20 system and UV detector (Shimadzu Corp., Kyoto, Japan) interfaced to an API 3200 Q-trap instrument with a TurboIonSpray interface, from AB Sciex (Concord, ON, Canada) was used. BPDE-alkylated peptide analytes were analysed by two different LC setup conditions; setup 1 was favoured for obtaining separation between the His-Pro analytes from (±)-*anti*-, (+)-*anti*- and (±)-*syn*-BPDE, and setup 2 was used for separation between the respective analytes of BPDE-His-Pro and BPDE-Lys. In setup 1 the LC was coupled directly to the MS/MS via a column, while in setup 2 the UV detector, set at 345 nm, was interfaced prior to the MS/MS. In both setups, a Kinetex C18 2.6 μm, 150 × 2.1 mm column (Phenomenex) was used and the mobile phase consisted of A, 0.1% (v/v) formic acid in methanol/water (5:95, v/v), and B, 0.1% (v/v) formic acid in methanol/water (95:5, v/v). For setup 1 gradient used was 5% B for 2 min, increased to 100% B in 24 min, at 100% B for 2 min, down to 5% B in 1 min and at 5% B for 10 min, with flow rate 0.2 mL/min, and injection volume of 5 μL. For setup 2, 0% B for 2 min, increased to 100% B in 13 min, at 100% B for 2 min, down to 0% B in 1 min and at 0% B for 10 min, with flow rate 0.1 mL/min, and injection volume of 2 μL. The mass spectrometer was operated with electrospray ionisation in positive ion mode (ESI^+^) using multiple reaction monitoring (MRM) mode, with parameters similar to that described earlier^21^. The MRM transitions used for BPDE-His-Pro were 555 > 253, 257 and 303, for BPDE-His-Pro-Tyr were 718 > 416, 257 and 303, and for BPDE-Lys were 449 > 257 and 303. Acquisition and processing of data from the mass spectrometer were performed using the Analyst software version 1.5 from AB Sciex.

The BPDE-BME derivative was analysed by LC-UV. An Ascentis Express F5 2.7 μm, 150 × 2.1 mm column (Supelco Analytical) from the LC system was coupled to the UV detector set at 345 nm. Eluent A was 0.1% (v/v) formic acid in acetonitrile/water (5:95, v/v), and B was 0.1% (v/v) formic acid in acetonitrile/water (95:5, v/v). The gradient was 10% B for 3 min, increased to 70% B in 8 min, then to 100% B in 1 min, at 100% B for 4 min, down to 10% B in 1 min and at 10% B for 5 min, with flow rate 0.2 mL/min, and injection volume of 2 μL.

### Estimation of BPDE-adduct levels

Adduct levels of BPDE-His-Pro and BPDE-Lys formed from incubation of (±)-*anti*-, (+)-*anti*- and (±)-*syn*-BPDE with hSA followed by enzymatic digestion and isolation, as described above, were estimated using the respective areas from LC-UV and incorporating them in equation (1) (cf. Westberg *et al*.^21^).


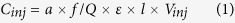


where, *C*_*inj*_, concentration in the injected samples (in M); *a*, peak area (microvolt second); *f*, flow rate (L/sec); *Q*, output voltage/absorption unit (microvolt per AU); *ε*, extinction coefficient (M^−1^cm^−1^); *l*, cuvette length (cm); and *V*_*inj*_, injection volume (L). Deoxyguanosine (dG) with *ε* 15400 M^−1^cm^−1^ at 259 nm was used as a reference to obtain Q, which is an instrument specific value calculated by analysing a compound with known extinction at a given concentration. The *ε* of BPDE-dG, 29000 M^−1^cm^−1^, at 345 nm was used as an approximate for the histidine and lysine adducts of BPDE. Influence on *ε* from the dG, His-Pro and Lys on the BPDE-adduct were considered to be negligible^21^.

### Calculation for dosimetry

Dose (*D*) of a reactive compound can be defined as the concentration (*C*) over time (*t*)^9^,^13^. *D* quantitatively takes into account the initial concentration (*C*_*0*_), disappearance rate (*λ*) and *t* according to equation (2). The further derivatised equation (3) was used for calculation of *D*. Subsequently, *D* and the measured adduct levels (*A*) were used for determining the rate for formation of SA adducts (*k*_*SA*_) as per equation (4).


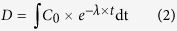



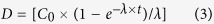



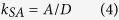


## Additional Information

**How to cite this article**: Motwani, H. V. *et al*. Interaction of benzo[a]pyrene diol epoxide isomers with human serum albumin: Site specific characterisation of adducts and associated kinetics. *Sci. Rep.* **6**, 36243; doi: 10.1038/srep36243 (2016).

**Publisher’s note:** Springer Nature remains neutral with regard to jurisdictional claims in published maps and institutional affiliations.

## Figures and Tables

**Figure 1:**
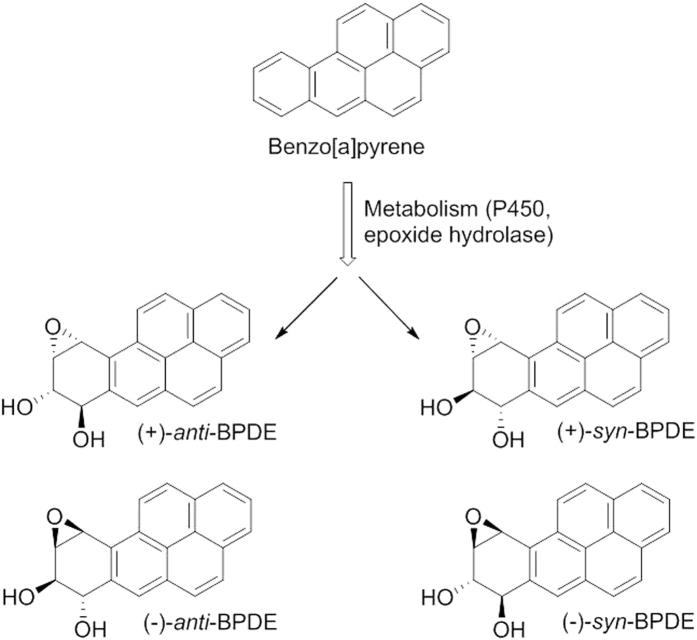
Benzo[a]pyrene and its four diol epoxide metabolites, the enantiomers (+)-*anti*-, (−)-*anti*-, (+)-*syn-* and (−)-syn-BPDE.

**Table 1:** Summary from LC-MS/MS analysis of BPDE-His-Pro and BPDE-Lys, formed from *in vitro* alkylation of hSA with (±)-*anti*-, (+)-*anti*- and (±)-*syn*-BPDE.

BPDE isomers	BPDE-His-Pro, Precursor ion m/z 555 [M+H]^+^			BPDE-Lys, Precursor ion m/z 449 [M+H]^+^		
	Retention time (min)^b^	Product ion (m/z)	Intensity	Retention time (min)^c^	Product ion (m/z)	Intensity
(±)-*anti*-	14.9/14.3	257	1.71×10^5^ au^d^	15.3/14.9	257	4.54×10^4^ au
		253	1.02×10^5^ au		253	n.d.
		303	1.13×10^4^ au		303	2.31×10^3^ au
(+)-*anti*-	14.3	257	14%^e^	15.3/14.9	257	43%
		253	18%		253	n.d.
		303	17%		303	41%
(−)-*anti*-a	14.9	257	86%	15.3/14.9	257	57%
		253	82%		253	n.d.
		303	83%		303	59%
(±)-*syn*-	14.5 (broad)	257	6.06×10^4^ au	15.3/16.0	257	7.94×10^4^ au
		253	2.91×10^4^ au		253	n.d.
		303	3.85×10^3^ au		303	4.39×10^3^ au

^a^(−)-*anti*-BPDE values were estimated based on the racemic (±)-*anti-* and enantiomer (+)-*anti*-BPDE.

^b^LC system setup 1.

^c^LC system setup 2.

^d^area, measured as arbitrary units by the Analyst software used for quantification.

^e^% of (±)-*anti*-BPDE; n.d., not determined.

**Table 2:** Histidine- and lysine-adduct levels (*A*_*His-Pro*_and *A*_*Lys*_, respectively) from BPDEs, and disappearance rate (*λ*) of the epoxides.

BPDE isomers	*A*_*His-Pro*_ (pmol adduct/mg SA)	*A*_*Lys*_ (pmol adduct/mg SA)	BPDE *λ* (s^−1^ × 10^-5^) [*n*, *r*^*2*^]^b^
(±)-*anti*-	1.4	1.4	1.7 ± 0.1^c^ [9, 0.96]
(+)-*anti*-	0.28	2.2	24 ± 2 [6, 0.97]
(−)-*anti*-a	2.4	0.64	n.d.
(±)-*syn*-	1.3	1.9	19 ± 5 [6, 0.78]

^a^(−)-*anti*-BPDE values were estimated based on the racemic (±)-*anti-* and enantiomer (+)-*anti*-BPDE.

^b^Number of time points *n* and correlation coefficient as *r*^*2*^ are given in parenthesis (cf. Fig. 5).

^c^SD calculated using LINEST; n.d., not determined.

**Table 3:** Dose (*D*) of BPDEs in the hSA mixture and rate constants for reactions with histidine (*k*_*His-Pro*_) and lysine (*k*_*Lys*_).

BPDE isomers	*D* (mM·h)	*k*_*His-Pro*_ (L·g^−1^·h^−1^)	*k*_*Lys*_ (L·g^-1^·h^-1^)
(±)-*anti*-	0.83	1.7 × 10^-6^	1.7 × 10^-6^
(+)-*anti*-	0.078	3.6 × 10^-6^	2.8 × 10^−5^
(−)-*anti*-a	1.6	n.d.	n.d.
(±)-*syn*-	0.099	1.3 × 10^−5^	1.9 × 10^−5^

^a^(−)-*anti*-BPDE values were estimated based on the racemic (±)-*anti-* and enantiomer (+)-*anti*-BPDE; n.d., not determined.

## References

[b1] Agency for Toxic Substances and Disease Registry (ATSDR), Toxicological profile for polycyclic aromatic hydrocarbons. US Department of Health and Human Services, Atlanta, GA (1995). 38091452

[b2] International Agency for Research on Cancer (IARC), A review of human carcinogens: chemical agents and related occupations. Monographs on the evaluation of carcinogenic risks to humans. Vol 100 F, Lyon, France (2012). PMC4781612 23189753

[b3] Boström C. E. . Cancer risk assessment, indicators, and guidelines for polycyclic aromatic hydrocarbons in the ambient air. Environ Health Perspect 110, 451–488 (2002). 12060843 10.1289/ehp.110-1241197 PMC1241197

[b4] European Food Safety Authority (EFSA). Polycyclic aromatic hydrocarbons in food - Scientific opinion of the panel on contaminants in the food chain. The EFSA Journal 724, 1–114 (2008). 10.2903/j.efsa.2008.653 PMC10193653 37213838

[b5] Abramsson-Zetterberg L., Darnerud P. O. & Wretling S. Low intake of polycyclic aromatic hydrocarbons in Sweden: results based on market basket data and a barbecue study. Food Chem Toxicol 74, 107–111 (2014). 25261863 10.1016/j.fct.2014.09.004

[b6] Sims P., Grover P. L., Swaisland A., Pal K. & Hewer A. Metabolic activation of benzo(a)pyrene proceeds by a diol-epoxide. Nature 252, 326–328 (1974). 4473724 10.1038/252326a0

[b7] Xue W. & Warshawsky D. Metabolic activation of polycyclic and heterocyclic aromatic hydrocarbons and DNA damage: a review. Toxicol Appl Pharmacol 206, 73–93 (2005). 15963346 10.1016/j.taap.2004.11.006

[b8] Buening M. K. . Tumorigenicity of the optical enantiomers of the diastereomeric benzo[a]pyrene 7,8-diol-9,10-epoxides in newborn mice: exceptional activity of (+)-7beta,8alpha-dihydroxy-9alpha,10alpha-epoxy-7,8,9,10-tetrahydrobenzo[a]pyrene. Proc Natl Acad Sci USA 75, 5358–5361 (1978). 281685 10.1073/pnas.75.11.5358 PMC392962

[b9] Törnqvist M. . Protein adducts: quantitative and qualitative aspects of their formation, analysis and applications. J Chromatogr B Analyt Technol Biomed Life Sci 778, 279–308 (2002). 10.1016/s1570-0232(02)00172-1 12376136

[b10] Lee B. M. . Immunologic measurement of polycyclic aromatic hydrocarbon-albumin adducts in foundry workers and roofers. Scandinavian Journal of Work, Environment & Health 17, 190–194 (1991). 10.5271/sjweh.1711 2068558

[b11] Fred C., Törnqvist M. & Granath F. Evaluation of cancer tests of 1,3-butadiene using internal dose, genotoxic potency, and a multiplicative risk model. Cancer Res 68, 8014–8021 (2008). 18829559 10.1158/0008-5472.CAN-08-0334

[b12] Granath F. N., Vaca C. E., Ehrenberg L. G. & Törnqvist M. Cancer risk estimation of genotoxic chemicals based on target dose and a multiplicative model. Risk Anal 19, 309–320 (1999). 10765407 10.1023/a:1006933913194

[b13] Ehrenberg L., Moustacchi E., Ostermangolkar S. & Ekman G. Dosimetry of genotoxic agents and dose-response relationships of their effects. Mutation Research 123, 121–182 (1983). 6353222 10.1016/0165-1110(83)90024-6

[b14] Fasano M. . The extraordinary ligand binding properties of human serum albumin. Iubmb Life 57, 787–796 (2005). 16393781 10.1080/15216540500404093

[b15] Jr Peters T. Serum albumin in Advances in Protein Chemistry. Vol. 37 161–245 (1985). 3904348 10.1016/s0065-3233(08)60065-0

[b16] Boysen G. & Hecht S. S. Analysis of DNA and protein adducts of benzo[alpha]pyrene in human tissues using structure-specific methods. Mutat Res-Rev Mutat 543, 17–30 (2003). 10.1016/s1383-5742(02)00068-6 12510015

[b17] Kafferlein H. U., Marczynski B., Mensing T. & Bruning T. Albumin and hemoglobin adducts of benzo[a]pyrene in humans-Analytical methods, exposure assessment, and recommendations for future directions. Crit Rev Toxicol 40, 126–150 (2010). 20085480 10.3109/10408440903283633

[b18] Day B. W., Skipper P. L., Zaia J., Singh K. & Tannenbaum S. R. Enantiospecificity of covalent adduct formation by benzo[a]pyrene anti-diol epoxide with human serum albumin. Chem Res Toxicol 7, 829–835 (1994). 7696539 10.1021/tx00042a017

[b19] Chung M. K., Regazzoni L., McClean M., Herrick R. & Rappaport S. M. A sandwich ELISA for measuring benzo[a]pyrene-albumin adducts in human plasma. Anal Biochem 435, 140–149 (2013). 23333225 10.1016/j.ab.2012.12.021 PMC6354764

[b20] Helleberg H. & Tornqvist M. A new approach for measuring protein adducts from benzo[a]pyrene diolepoxide by high performance liquid chromatography/tandem mass spectrometry. Rapid Commun Mass Spectrom 14, 1644–1653 (2000). 10962485 10.1002/1097-0231(20000930)14:18<1644::AID-RCM74>3.0.CO;2-#

[b21] Westberg E. . Conditions for sample preparation and quantitative HPLC/MS-MS analysis of bulky adducts to serum albumin with diolepoxides of polycyclic aromatic hydrocarbons as models. Anal Bioanal Chem 406, 1519–1530 (2014). 24390408 10.1007/s00216-013-7540-7

[b22] Brunmark P. . Identification of subdomain IB in human serum albumin as a major binding site for polycyclic aromatic hydrocarbon epoxides. Chem Res Toxicol 10, 880–886 (1997). 9282837 10.1021/tx9700782

[b23] Kesner L., Muntwyler E. & Geriffin G. Studies on the hydrolytic action of pronase on derivatized and non-derivatized proteins. Biochim Biophys Acta 85, 435–440 (1964). 14194858 10.1016/0926-6569(64)90307-4

[b24] Westberg E. A. C. . Adduct levels from benzo[a]pyrenediol epoxide: Relative formation to histidine in serum albumin and to deoxyguanosine in DNA *in vitro* and *in vivo* in mice measured by LC/MS-MS methods. Toxicology Letters 232, 28–36 (2015). 25261590 10.1016/j.toxlet.2014.09.019

[b25] Islam G. A., Greibrokk T., Harvey R. G. & Ovrebo S. HPLC analysis of benzo[a]pyrene-albumin adducts in benzo[a]pyrene exposed rats. Detection of cis-tetrols arising from hydrolysis of adducts of anti- and syn-BPDE-III with proteins. Chem Biol Interact 123, 133–148 (1999). 10597906 10.1016/s0009-2797(99)00129-5

[b26] Islam N. B., Whalen D. L., Yagi H. & Jerina D. M. Kinetic studies of the reactions of benzo[a]pyrene-7,8-diol 9,10-epoxides in aqueous solutions of human serum albumin and nonionic micelles. Chem Res Toxicol 1, 398–402 (1988). 2979757 10.1021/tx00006a012

[b27] Whalen D. L., Montemarano J. A., Thakker D. R., Yagi H. & Jerina D. M. Changes of mechanisms and product distributions in hydrolysis of benzo[a]pyrene-7,8-diol 9,10-epoxide metabolites induced by changes in pH. J Am Chem Soc 99, 5522–5524 (1977). 18500 10.1021/ja00458a069

[b28] Roche C. J., Zinger D., Geacintov N. E. & Harvey R. G. Enhancement of stability of 7 beta,8 alpha-dihyroxy-9 alpha epoxybenzo(a)pyrene by complex formation with serum albumin. Cancer Biochem Biophys 8, 35–40 (1985). 3928138

[b29] Skipper P. L. Influence of tertiary structure on nucleophilic substitution reactions of proteins. Chem Res Toxicol 9, 918–923 (1996). 8870977 10.1021/tx960028h

[b30] Rubino F. M., Pitton M., Di Fabio D. & Colombi A. Toward an “omic” physiopathology of reactive chemicals: Thirty years of mass spectrometric study of the protein adducts with endogenous and xenobiotic compounds. Mass Spectrom Rev 28, 725–784 (2009). 19127566 10.1002/mas.20207

[b31] Chung M. K. . A sandwich enzyme-linked immunosorbent assay for adducts of polycyclic aromatic hydrocarbons with human serum albumin. Anal Biochem 400, 123−129 (2010). 20083082 10.1016/j.ab.2010.01.018 PMC2842209

[b32] Zaia J. & Biemann K. Characteristics of high energy collision-induced dissociation tandem Mass Spectra of Polycyclic aromatic Hydrocarbon diolepoxide adducted peptides. J Am Soc Mass Spectrom 5, 649–654 (1994). 24221967 10.1016/1044-0305(94)85006-2

[b33] Vikström A. C. . *In vivo* doses of acrylamide and glycidamide in humans after intake of acrylamide-rich food. Toxicol Sci 119, 41–49 (2011). 20952504 10.1093/toxsci/kfq323

[b34] Motwani H. V. & Törnqvist M. *In vivo* doses of butadiene epoxides as estimated from *in vitro* enzyme kinetics by using cob(I)alamin and measured hemoglobin adducts: An inter-species extrapolation approach. Toxicol Appl Pharm 281, 276–284 (2014). 10.1016/j.taap.2014.10.011 25448046

[b35] Michaud D. P., Gupta S. C., Whalen D. L., Sayer J. M. & Jerina D. M. Effects of pH and salt concentration on the hydrolysis of a benzo[alpha]pyrene 7,8-diol-9,10-epoxide catalyzed by DNA and polyadenylic acid. Chem Biol Interact 44, 41–52 (1983). 6406081 10.1016/0009-2797(83)90128-x

